# Supplementary material for: UBA3 reduction sensitizes cancer cells to NAE inhibitors
Source: Life Sci Alliance. 2026 Apr 29;9(7):e202503589. doi: 10.26508/lsa.202503589 (PMC13129363; doi:10.26508/lsa.202503589)
Supplement: Supplementary file 2 [file LSA-2025-03589_TableS1.docx]

**Table.S1. A retrospective analysis on the differential SOMCL-19-133 sensitivity of cancer cells with low and high expression of *UBA3* mRNA.**

| **Cell lines** | **Cancer types** | ***UBA3* mRNA (nPTM)** | **IC_50_/SOMCL-19-133 (nM)** |
| --- | --- | --- | --- |
| **Low expression of *UBA3* mRNA** | | | |
| SUIT-2 | Pancreatic cancer | 31 | 29.22 |
| NCI-H1975 | Lung cancer | 31 | 108.47 |
| SW48 | Colon cancer | 32 | 5.59 |
| Panc 02.03 | Pancreatic cancer | 37 | 97.25 |
| NCI-H226 | Lung cancer | 38 | 124.18 |
| Jurkat | Leukemia | 40 | 70.43 |
| Average | - | 35 | 72.52 |
| **High expression of *UBA3* mRNA** | | | |
| PC-3 | Prostate cancer | 42 | 962.55 |
| HCT-116 | Colon cancer | 47 | 15.71 |
| NCI-N87 | Gastric cancer | 49 | 65.08 |
| TOV-112D | ovarian cancer | 52 | 66.00 |
| HT-29 | Colon cancer | 54 | 40.22 |
| SW620 | Colon cancer | 57 | 2164.76 |
| DU145 | Prostate cancer | 60 | 12.55 |
| THP-1 | Leukemia | 60 | 33.59 |
| HCT-15 | Colon cancer | 61 | 56.88 |
| Capan-1 | Pancreatic cancer | 63 | 9.44 |
| NOMO-1 | Leukemia | 64 | 159.29 |
| Miapaca-2 | Pancreatic cancer | 68 | 17.98 |
| OVCAR-8 | Ovarian cancer | 70 | 127.15 |
| HGC-27 | Gastric cancer | 71.3 | 22.03 |
| RKO | Colon cancer | 73 | 40.23 |
| LNCaP | Prostate cancer | 74 | 15.24 |
| MV-4-11 | Leukemia | 75 | 40.83 |
| SW480 | Colon cancer | 83 | 732.48 |
| KG-1 | Leukemia | 87 | 204.37 |
| Average | - | 64 | 251.91 |
| H/L (fold) | - | 1.83 | 3.47 |

Notes: UBA3 mRNA values were from the Human Protein Atlas (https://www. proteinatlas.org/); the IC_50_ values of SOMCL-19-133 from Reference 2 were presented as Mean.
