# Supplementary material for: UBA3 reduction sensitizes cancer cells to NAE inhibitors
Source: Life Sci Alliance. 2026 Apr 29;9(7):e202503589. doi: 10.26508/lsa.202503589 (PMC13129363; doi:10.26508/lsa.202503589)
Supplement: Supplementary file 3 [file LSA-2025-03589_TableS2.docx]

**Table.S2. Differential SOMCL-19-133 sensitivity of cancer cells**

**with low and high expression of *UBA3* mRNA.**

| **Cell lines** | **Cancer types** | ***UBA3* mRNA (nPTM)** | **IC_50_/SOMCL-19-133 (nM)** |
| --- | --- | --- | --- |
| Low expression of *UBA3* mRNA | | | |
| KYSE-270 | Esophageal cancer | 13.90 | 68.62±4.11 |
| MCF-7 | Breast cancer | 25.7 | 34.44 ± 0.40 |
| T47-D | Breast cancer | 33 | 42.39 ± 4.49 |
| NCI-N87 | Gastric cancer | 33.2 | 38.15 ± 2.25 |
| LoVo | Colorectal cancer | 35.00 | 59.44 ± 6.85 |
| DMS-114 | Lung cancer | 36.10 | 3.91 ± 1.59 |
| CAL27 | Head and neck cancer | 39.9 | 12.99 ± 1.89 |
| Fadu | Head and neck cancer | 39 | 35.99 ± 2.80 |
| Average | - | 32 | 36.99 |
| High expression of *UBA3* mRNA | | | |
| SW620 | Colorectal cancer | 54.7 | 2164.76 ± 221.23 |
| BT-549 | Breast cancer | 55.9 | 277.64 ± 79.11 |
| SK-OV-3 | Ovarian cancer | 61.5 | 211.12±25.85 |
| AGS | Gastric cancer | 64 | 242.14 ± 67.77 |
| KYSE-70 | Esophageal cancer | 71 | 285.01 ± 6.03 |
| NCI-H82 | Lung cancer | 72.6 | 231.03 ± 69.02 |
| MDA-MB-231 | Breast cancer | 76.5 | 1390.78 ± 886.58 |
| VCAP | Prostrate cancer | 81.9 | 227.26 ± 59.95 |
| Average | - | 67 | 628.72 |
| H/L (fold) | - | 2.09 | 17.00 |

Notes: *UBA3* mRNA values were from the Human Protein Atlas (https://www. proteinatlas.org/); the IC_50_ values of SOMCL-19-133 from 3 independent experiments (SRB assays) were presented as Mean±SD.
