# Supplementary material for: UBA3 reduction sensitizes cancer cells to NAE inhibitors
Source: Life Sci Alliance. 2026 Apr 29;9(7):e202503589. doi: 10.26508/lsa.202503589 (PMC13129363; doi:10.26508/lsa.202503589)
Supplement: Supplementary file 4 [file LSA-2025-03589_TableS3.docx]

**Table.S3. The nucleotide sequences used in this study.**

| **Names** | | | **siRNA sequences** | | | |
| --- | --- | --- | --- | --- | --- | --- |
|  |  |  | **Sense** | | **Antisense** | |
| siNAE1-1 | | | 5’-GCAGAUUCAGGCAAAUAUATT-3’ | | | 5’-UAUAUUUGCCUGAAUCUG  CTT-3’ |
| siNAE1-2 | | | 5’-GCUCGUGCCUUAAAGGAA  UTT-3’ | | | 5’-AUUCCUUUAAGGCACGAGCTT-3’ |
| siNAE1-3 | | | 5’-GCUCAUGUUUGCCUAAUA ATT-3’ | | | 5’-UUAUUAGGCAAACAUGAGCTT- 3’ |
| siUBA3-1 | | | 5’-GCUGAUAUCUCUUCUAAA  UTT-3’ | | | 5’-AUUUAGAAGAGAUAUCAGCTT-3’ |
| siUBA3-2 | | | 5’-GUCGCUGGAACCAUGUAAATT-3’ | | | 5’-UUUACAUGGUUCCAGCGACTT-3’ |
| siUBA3-3 | | | 5’-GAGAGCAUCACAAUAUAAUTT-3’ | | | 5’-AUUAUAUUGUGAUGCUCUCTT3’ |
| **Names** | | | **shRNA sequences** | | | |
|  |  |  | **Forward** | | **Reverse** | |
| shNAE1_1 | | | 5’-CCGGGCCATGGAATTCTTACAAGAACTCGAGTTCTTGTAA GAATTCCATGGCTTTTT-3’ | | | 5’- AATTAAAAAGCCATGGAA TTCTTACAAGAACTCGAGTTCTTGTAAGAATTCCATGGC-3’ |
| shNAE1_2 | | | 5’-CCGGCCAGGAGTATCTAACTATCAACTCGAGTTGATAGTTAGATACTCCT GGTTTTT-3’ | | | 5’-AATTAAAAACCAGGAGTATCTAACTATCAACTCGAGTTGATAGTTAGATACTCCTGG-3’ |
| shNAE1_3 | | | 5’-CCGGGCATGTCACAAACTTCAGCAACTCGAGTTGCTGAAGTTTGTGACATGCTTTTT-3’ | | | 5’-AATTAAAAAGCATGTCACAAACTTCAGCAACTCGAGTTGCTGAAGTTTGTGACATGC-3’ |
| shUBA3_1 | | 5’-CCGGCGACACTTTCTATCGACAATTCTCGAGAATTGTCGATAGAAAGTGTCGTTTTT-3’ | | | | 5’-AATTAAAAACGACACTTTCTATCGACAATTCTCGAGAATTGTCGATAGAAAGTGTCG-3’ |
| shUBA3_2 | | 5’-CCGGCCACAGACTGTACTATTCAAACTCGAGTTTGAATAGTACAGTCTGTGGTTTTT-3’ | | | | 5’-AATTAAAAACCACAGACTGTACTATTCAAACTCGAGTTTGAATAGTACAGTCTGTGG-3’ |
| shUBA3_3 | | 5’-CCGGCCTCTATTGAAGAACGAACAACTCGAGTTGTTCGT TCTTCAATAGAGGTTTTT-3’ | | | | 5’-AATTAAAAACCTCTATTGAAGAACGAACAACTCGAGTTGTTCGTTCTTCAATAGAGG-3’ |
| **Names** | | **sgRNA sequences** | | | | |
|  |  | **Forward** | | | | **Reverse** |
| sgUBA3-1 | | 5’-CACCGTACATGTATCTAACAAGAAC-3’ | | | | 5’-AAACGTTCTTGTTAGATACATGTAC-3’ |
| sgUBA3-2 | | 5’-CACCGAAAGTTCTAGTCATTGGAGC-3’ | | | | 5’- AAACGCTCCAATGACTAG AACTTTC-3’ |
| sgUBA3-3 | | 5’-CACCGACCTCCTGTAGTTTAGCTGA-3’ | | | | 5’-AAACTCAGCTAAACTACAGGAGGTC-3’ |
| **Names** | | **Primer sequences for RT-qPCR** | | | | |
|  |  | **Forward** | | | | **Reverse** |
| NAE1 | | 5’-TGGATGCTCAGCAAACAAAAAC-3’ | | | | 5’-TTTCAGTTCCTGTGGCTGTTG-3’ |
| UBA3 | | 5’-GGATGGCGAGGAGCCAA  T-3’ | | | | 5’-TGGAGAGATTCAGTGCTCGGT-3’ |
| GAPDH | | | 5’-TCCAAAATCAAGTGGGGCGA-3’ | | 5’-AAATGAGCCCCAGCCTTC  TC-3’ | |
